# Improving Drug Delivery for Alzheimer’s Disease Through Nose-to-Brain Delivery Using Nanoemulsions, Nanostructured Lipid Carriers (NLC) and in situ Hydrogels

**DOI:** 10.2147/IJN.S305851

**Published:** 2021-06-29

**Authors:** Sara Cunha, Ben Forbes, José Manuel Sousa Lobo, Ana Catarina Silva

**Affiliations:** 1 UCIBIO/REQUIMTE, MEDTECH Laboratory of Pharmaceutical Technology, Department of Drug Sciences, Faculty of Pharmacy, University of Porto, Porto, 4050-313, Portugal; 2 Institute of Pharmaceutical Science, Faculty of Life Sciences and Medicine, King’s College London, London, SE1 9NH, UK; 3 UFP Energy, Environment and Health Research Unit (FP ENAS), Fernando Pessoa University, Porto, 4249-004, Portugal

**Keywords:** Alzheimer’s disease, AD, nose-to-brain delivery, nanoemulsions, nanostructured lipid carriers, NLC, in situ hydrogels

## Abstract

Current treatments for Alzheimer’s disease (AD) attenuate the progression of symptoms and aim to improve the patient’s quality of life. Licensed medicines are mostly for oral administration and are limited by the difficulty in crossing the blood–brain barrier (BBB). Here in, the nasal route has been explored as an alternative pathway that allows drugs to be directly delivered to the brain via the nasal cavity. However, clearance mechanisms in the nasal cavity impair the delivery of drugs to the brain and limit their bioavailability. To optimize nose-to-brain delivery, formulations of lipid-based nanosystems, namely nanoemulsions and nanostructured lipid carriers (NLC), formulated in situ gelling hydrogels have been proposed as approaches for nose-to-brain delivery. These formulations possess characteristics that facilitate drug transport directly to the brain, minimizing side effects and maximizing therapeutic benefits. It has been recommended that the manufacture of these drug delivery systems follows the quality by design (QbD) approach based on nasal administration requirements. This review provides an insight into the current knowledge of the AD, highlighting the need for an effective drug delivery to the brain. Considering the mounting interest in the use of nanoemulsions and NLC for nose-to-brain delivery, a description of drug transport pathways in the nasal cavity and the application of these nanosystems and their in situ hydrogels through the intranasal route are presented. Relevant preclinical studies are summarised, and the future prospects for the use of lipid-based nanosystems in the treatment of AD are emphasized.

## Introduction

Alzheimer’s disease (AD) is one of the most prominent neurodegenerative disorders worldwide that is associated with severe dementia. It represents a public health problem, as there is currently no cure. AD is initially characterized by short-term memory loss that proceeds to more severe deficits due to neuronal damage.^1^,^2^ A global estimate suggests that 131.5 million people will live with the disease in 2050, as the number of cases since 2015 is progressively increasing.^1^ Even though AD’s pathogenesis is not completely clear, the aggregation of misfolded tau proteins that lead to the formation of intraneuronal neurofibrillary tangles, extracellular senile plaques, neuronal loss, and activation of microglia have been considered the main hallmarks of the disease.^3^,^4^ More profound knowledge has indicated that multiple factors related to genetic alterations, innate immune responses, systemic neuronal inflammation, aging, and an unbalanced diet could lead to widespread neuronal degeneration, synaptic loss, and diffuse brain atrophy.^3^,^5^,^6^ Herein, neuroinflammation has emerged as a critical factor in AD.^7^ The current treatment of AD reduces the progression of symptoms and improves mental and physical disability, using pharmacological and non-pharmacological treatments. Pharmacological treatment includes long-term monotherapy acetylcholinesterase (AChE) inhibitors or dual combination of AChE inhibitors and N-methyl-d-aspartate (NMDA) receptor antagonists. These drugs are administered through the oral and transdermal routes, entering to the systemic circulation and undergoing metabolic degradation, which decreases bioavailability. In addition, these drugs need to cross the blood–brain barrier (BBB) to reach the central nervous system (CNS), which is difficult for high molecular weight and hydrophilic molecules.^8^,^9^ Among non-pharmacological treatments, bioactive compounds found in fruit and vegetables have a promising role in protecting and delaying the progression of AD. Examples of these compounds include fat-soluble vitamins, carotenoids, phenolic compounds, omega-3 fatty acids, and isothiocyanates, which have antioxidant and anti-inflammatory effects and modulate the formation of amyloid plaques and tau tangles.^10^,^11^

Nasal drug administration is an established alternative to other administration routes, for non-invasive systemic and local nasal drug delivery.^12^,^13^ Additionally, the nasal route is a pathway for nose-to-brain delivery, allowing the direct delivery of the drug to the brain; moreover, it avoids first-pass effect and the need for transport across the BBB.^14^,^15^ Several pharmaceutical dosage forms have been developed for intranasal drug delivery, including powders, nasal sprays, in situ hydrogels, and formulations containing nanosystems.^13^,^14^

In recent decades, nanosystems’ development has been at the top of the list of priorities of drug delivery researchers.^16^ In this field, lipid-based nanosystems, such as nanoemulsions and nanostructured lipid carriers (NLC), have been identified as efficient systems to deliver lipophilic drugs, protecting them from elimination in the nasal cavity by enzymes and mucociliary clearance. Surface modification with biomolecules like proteins and antibodies and the use of thermosensitive and mucoadhesive polymers to develop in situ hydrogel matrices are being exploited to enhance lipid-based nanosystem therapeutic potential and reduce adverse effects.^16^,^17^

To develop lipid-based nanosystems that fulfil the requisites of nasal delivery, regulatory entities have required the use of the quality by design (QbD) approach to produce formulations with high quality, safety, and efficacy, based on quality risk management that avoids the risk of failure.^18–20^ The manufacturing process for lipid-based nanosystem formulations begins with the design of experiment (DoE), considering the critical quality attributes (CQAs) essential for the fulfilment of the quality target product profile (QTPP). Thereafter, quality should be monitored during the whole product lifecycle using a control strategy.^21^,^22^ For nose-to-brain delivery, important QTPPs are small particle size (100–200 nm), narrow polydispersity index (PDI) (0.2–0.3), high zeta potential (ZP) (~|30 mV|), isotonic (230–320 mOsm/kg), pH (5.5–6.0) and adhesion to the nasal mucosa.^21–23^

This review provides an insight into the current knowledge of the pathogenesis and treatments associated with AD, highlighting the need to exploit new drug delivery routes, such as intranasal administration of drugs for delivery to the brain. The advantages of using lipid-based nanosystems for nasal drug administration for AD treatment are evaluated, and preclinical studies with nanoemulsions, NLC, and in situ hydrogels are discussed.

## Pathogenesis of Alzheimer’s Disease

In recent decades, a number of hypotheses have been investigated to explain AD pathogenesis. Notable among these are the cholinergic hypothesis, the amyloid cascade hypothesis, and the oxidative stress and ApoE hypothesis. More recently, the role of systemic inflammation and neuroinflammation has attracted attention.

### Cholinergic Hypothesis

The cholinergic hypothesis postulates that AD is caused by a reduction in acetylcholine synthesis in the brain. It assumes that, in the early stage of AD, the brain has low levels of choline acetyltransferase, and cholinergic neurons involved in the synthesis of acetylcholine lose their function, resulting in cognitive dysfunction.^4^,^24^ Thus, a widely studied therapeutic strategy in AD is to increase cholinergic levels in the brain by inhibiting its degradation using AChE inhibitors.^25^,^26^ However, the cholinergic hypothesis has been questioned, since AChE inhibitors do not stop AD’s progression, providing only relief of cognitive symptoms.^4^,^25^

### Amyloid Cascade Hypothesis

The amyloid cascade hypothesis suggests that the aggregation of pathological forms of the amyloid beta-peptide (Aβ) may be responsible for neuropathologies, including the occurrence of neurofibrillary tangles. The discovery of genetic mutations associated with AD that occur in presenilin 1 and presenilin 2 (PSEN1 and PSEN2) supports this hypothesis and gives rise to alterations in the proteolytic processing of amyloid precursor protein (APP), with consequent overexpression of abnormal Aβ species. These findings are consistent with excessive Aβ production by APP overexpression being involved in the pathogenesis of AD.^27^,^28^

### Oxidative Stress and ApoE Hypothesis

Oxidative stress is a disturbance in the production of toxic reactive oxygen species (ROS) associated with an imbalance in the production of antioxidants through the glutathione system and the repair functions of deoxyribonucleic acid (DNA).^4^,^29^ Mitochondria have been described as the main organelle involved in oxidative stress as their dysfunction causes ROS production. Oxidative stress has been associated with neurodegenerative disorders, since neuronal cells are vulnerable to free radical damage.^30–32^ ApoE is a lipoprotein produced mainly by astrocytes with receptors belonging to the family of low-density lipoprotein (LDL) genes involved in cholesterol transport.^4^ The ApoE gene is located on chromosome 19 and has three types of polymorphic alleles, ie, ApoE2 (ε2), ApoE3 (ε3), and ApoE4 (ε4), and is the main genetic risk factor associated with late-onset AD.^4^,^33^,^34^ ApoE4 can cause AD by reducing Aβ clearance, increasing Aβ aggregation or through other mechanisms, such as neuroinflammation, tauopathy and decreasing glucose metabolism in the brain. Targeting ApoE may be a future strategy for managing AD.^4^,^34^

### Neuroinflammation

Neurodegeneration has been associated with changes in the immune system that cause failure of innate and adaptive immune responses in the CNS and an imbalance in the regeneration system.^4^,^7^,^35^ This process occurs when the microglia and macrophages lose their ability to phagocytose inflammatory agents, such as pollutants, metals, and toxic compounds which activate the innate pathogenic immune responses and stimulate the formation of protein aggregates related to neurodegenerative diseases.^35^,^36^ Activation of microglia occurs when Aβ oligomers bind to receptors on the microglia cell surface, leading to the production and release of proinflammatory cytokines and chemokines, such as TNF-α and IL-1β.^37–39^ It is known that the progression of AD is related to a decline in the microglia’s phagocytic activity and in an increase in the levels of proinflammatory cytokines and neurotoxic molecules. Although Aβ deposition may give rise to an inflammatory process by itself, traumatic brain injury, obesity, and systemic inflammation may provide a sustained neuroinflammatory stimulus that promotes the development of AD.^5^,^40^

### Systemic Inflammation

**Figure 1:**
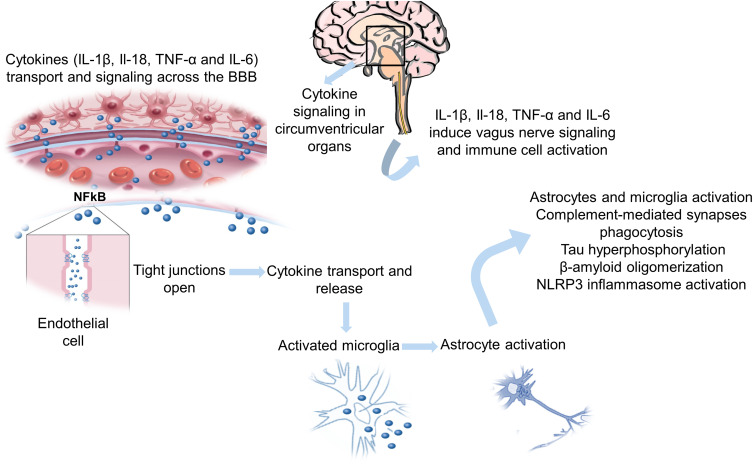
Schematic representation of the systemic process of inflammation in AD neuroinflammation.

**Notes:** Adapted with permission from: Walker KA, Ficek BN, Westbrook R. Understanding the Role of Systemic Inflammation in Alzheimer’s Disease. *ACS Chem Neurosci*. 2019;10(8):3340–3342.^40^ Copyright © 2019 American Chemical Society. And from: Focused Ultrasound Foundation. Blood-Brain Barrier Opening. Available at: https://www.fusfoundation.org/mechan isms-of-action/blood-brain-barrier-opening; 2019 [cited 15 July 2020].^113^ © 1999–2021 Focused Ultrasound Foundation. Use of the image courtesy of the Focused Ultrasound Foundation.

AD may be considered a systemic disease since it comprises inflammation in the brain and inflammatory reactions in the periphery.^41^ Cytokines are produced during systemic inflammation that can cross the BBB and signal to the CNS through the glial barrier by stimulating the vagus nerve. These cytokines can connect with receptors on the surface of endothelial cells, leading to a cascade of signaling that acts in three directions (Figure 1). 3,6 i) the transcription factor NFκB can induce the release of cytokines into the systemic circulation, which can open the tight junctions of the endothelial cells; ii) vagus nerve stimulation by proinflammatory cytokines and endotoxins promotes the stimulation of the hypothalamic-pituitary-adrenal (HPA) axis; iii) vagus nerve causes the glutamatergic stimulation of neural-immune cells, inducing the release of proinflammatory cytokines and chemokines, which activate astrocytes and microglia to release proinflammatory cytokines. The overloading of kinases that induce tau hyperphosphorylation, complement-mediated synapse phagocytosis, β-amyloid oligomerization, as well as stimulation of the NLRP3 inflammasome, are activated by the continuous inflammatory process of microglia and astrocytes, causing neurodegeneration.^3^,^6^,^40^,^42^

## Treatment of Alzheimer’s Disease

### Challenges of Drug Delivery to the Central Nervous System

Currently, potential treatments for AD are hindered by the physiological and anatomical characteristics of the CNS.^43^,^44^ The CNS has three physiological barriers that are difficult for drugs to overcome:^45^ (i) the BBB that is formed by capillary endothelial cells and joined by tight junctions; (ii) the blood-leptomeningeal barrier (BLMB) that contacts with the cerebrospinal fluid (CSF) and has an unfenestrated endothelium connected by tight junctions; (iii) the blood-cerebrospinal fluid (blood-CSF) barrier that is composed by endothelial cells of the plexuses choroidal blood vessels that are connected by tight junctions.^46^ The BBB has low permeability, and the passage of molecules depends on their physicochemical characteristics and interaction with endogenous efflux transporters, ie, ATP binding cassette transporters. It is known that BBB limits the entry into the brain of about 98% of low molecular weight drugs and 100% of high molecular weight drugs.^47^ Thus, BBB is the main barrier to the transport of drugs from the systemic circulation to the CNS, decreasing bioavailability in the brain and leading to unsuccessful treatment of neurodegenerative diseases.^48^ Various strategies that enhance the passage of drugs across the BBB and its delivery into the brain have been studied,^47^,^49^,^50^ although with limited success.

### Conventional Treatment

The current AD treatment aims to reduce the disease symptoms progression, maintain patient’s quality of life, and delay cognitive decline.^8^,^9^ There is evidence that a combination of pharmacological and non-pharmacological treatments reduces the clinical progression and relieves symptoms, attenuating the progressive loss of cognitive and functional skills.^8^ Initially, the Food and Drug Administration (FDA) approved long-term monotherapy with an AChE inhibitor. However, the benefits of a dual combination treatment with AChE inhibitors and NMDA receptor antagonists have been recognized, and this combination is currently recommended.^8^,^51^ Other treatment strategies related to the tau and amyloid hypotheses include immunotherapy-based strategies that had unsuccessful results in Phase II or III of clinical trials or are still undergoing development.^51^,^52^ The subsequent failures in the development of preventive and modifying treatments have made AChE inhibitors the primary therapy in the management of symptoms and, perhaps, in decreasing the progression rate of AD.

The AChE inhibitors approved by the FDA and European Medicines Agency (EMA) are donepezil, commercialized as Aricept^®^ available in tablets (5, 10 and 23 mg);^53^ galantamine commercialized as Reminyl^®^ and Razadyne^®^ commercialized in tablets and oral solution (4, 8 and 12 mg), and capsules (8, 16 and 24 mg);^54^,^55^ and rivastigmine commercialized as Prometax^®^ in the form of tablets (1.5 mg, 3 mg, 45 mg, 6 mg),^56^ and as Exelon^®^ available in capsules (1.5 mg, 3 mg, 4.5 mg, and 6 mg) oral solution (2mg/mL) and as a transdermal patch (4.6 mg, 9.5 mg, and 13.3 mg).^57^,^58^ NMDA receptor antagonists approved by the FDA and European Medicines Agency (EMA) are commercialized as Ebixa^®^ available in tablets (5 mg, 10 mg, 15 mg, and 20 mg) and oral solution (5mg/pump actuation)^59^ and as Axura^®^ commercialized only in tablets (5 mg, 10 mg, 15 mg, and 20 mg).^60^

#### Acetylcholinesterase Inhibitors

AChE inhibitors prevent acetylcholine cleavage in the synapse, increasing the post-synaptic activation of nicotinic and muscarinic receptors.^25^,^26^ Acetylcholine is hydrolyzed in the brain by two enzymes, namely AChE and butyrylcholinesterase (BChE).^26^

Galantamine and donepezil are AChE inhibitors, while rivastigmine is an AChE and BChE inhibitor used in the symptomatic treatment of mild to moderate AD.^51^ Donepezil has high selectivity for AChE compared with BChE. It can reduce the early expression of inflammatory cytokines, inhibit glutamate excitotoxicity, reduce oxidative stress, and stimulate an AChE isoform production with a neuroprotective effect.^9^,^25^,^26^,^51^ Galantamine is also capable of binding to nicotinic cholinergic receptors and is valuable in treating AD’s cognitive symptoms.^8^,^26^ In 2000, rivastigmine was accepted as a new AChEs inhibitor to treat mild to moderate AD stages.^26^,^56–58^ Its precise mechanism of action is not yet entirely clear. Still, it inactivates AChE for a prolonged period through a carbamate region that attaches to AChE for a more extended time than the acetate region in acetylcholine hydrolysis, inactivating the enzyme.^8^,^26^,^61^ AChE inhibitor therapy is limited by side effects related to pharmacology in the gastrointestinal tract.^61^

#### N-Methyl-D-Aspartate Receptor Antagonists

NMDA receptors have a crucial role in synaptic plasticity, cognitive functions, and the establishment of long-term memory.^51^,^62^ Memantine was approved in 2002 as a non-competitive antagonist with a low-to-moderate affinity to NMDA receptors. The main side effects are dizziness, agitation, confusion, headache, diarrhoea, and constipation. Currently, there are no other NMDA receptor antagonists licensed for use in the treatment of AD.^51^

### New Therapeutic Agents

The development of novel therapeutic agents is a challenge, mainly because of the difficulty in designing clinical trials to study the effects of drugs on AD progression, since clinical benefits must be demonstrated in terms of cognitive performance.^51^,^63^ New therapeutic agents that are under development include beta-site amyloid precursor protein cleaving enzyme 1(BACE-1), glycogen synthase kinase type 3 (GSK-3β), monoamine oxidase inhibitors (MAOs), phosphodiesterases, and the human monoclonal anti-amyloid antibody aducanumab.^51^,^63^,^64^

### Non-Pharmacological Treatment

Non-pharmacological treatment has a crucial role in preventing and delaying the progress of AD. A balanced diet, rich in bioactive compounds, such as fat-soluble vitamins, carotenoids, phenolic compounds, omega-3 fatty acids, and isothiocyanates, has been considered crucial in AD, preventing oxidative stress and inflammation, which can cause neurodegeneration.^11^,^65^ Increasing evidence from in vitro and in vivo studies has shown that the main beneficial effects of bioactive compounds in AD are as follows:^10^,^11^,^66^ defense against oxidative stress; anti-inflammatory activity; inhibition of neuronal apoptosis; reduction of tau phosphorylation; prevention of tau aggregation; repairing the damage caused by free radicals and regulating cell signaling pathways.

Examples of bioactive compounds reported as beneficial for AD include curcumin that shows antioxidant activity, reduces inflammatory process and decreases microglia activity;^67^ resveratrol shows antioxidant and anti-inflammatory effects able to maintain homeostasis and enhance mitochondrial function;^68^ quercetin has anti-inflammatory, antioxidant, and anti-apoptotic activity;^69^ naringenin is an antioxidant and cholinesterase inhibitor;^70^ vitamin D has neuroprotective activity, regulating the levels of neurotransmitters;^71^ vitamin E shows protection against the formation of Aß-induced tau phosphorylation.^72^

## Current Strategies to Improve the Treatment of Alzheimer’s Disease

### Nasal Drug Administration

The nasal route has been suggested as an alternative to the parenteral and oral routes, due to the possibility of non-invasive and easy drug administration.^12^,^13^ Local administration of drugs is used to treat the nasal cavity pathologies, including rhinitis, sinusitis, congestion, and allergic conditions.^12^,^73^ Systemic drug administration through the nasal route is also possible since the nasal mucosa is relatively permeable and has abundant blood perfusion, enabling rapid drug absorption to the bloodstream.^13^,^73^

**Figure 2:**
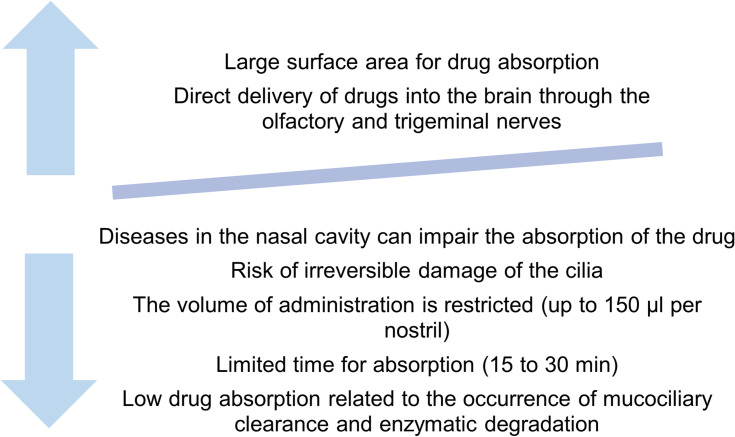
Main advantages and drawbacks of the nasal drug administration.

More recently, the nasal route has been extensively studied for the direct brain delivery of drugs through the nasal cavity, which presents a promising alternative, allowing drugs to be directly delivered into the brain, providing a means of bypassing the BBB and avoiding first-pass metabolism.^14^,^15^ Thereby, the nasal route has been considered a suitable alternative route of drug delivery for treatments for neurodegenerative diseases, such as AD, which requires drugs to be delivered to the brain.^74^,^75^ The main advantages and limitations of the nasal drug administration are described in Figure 2.^12^,^13^,^73^,^76–78^

### Transport Mechanisms of Nose-to-Brain Delivery

**Figure 3:**
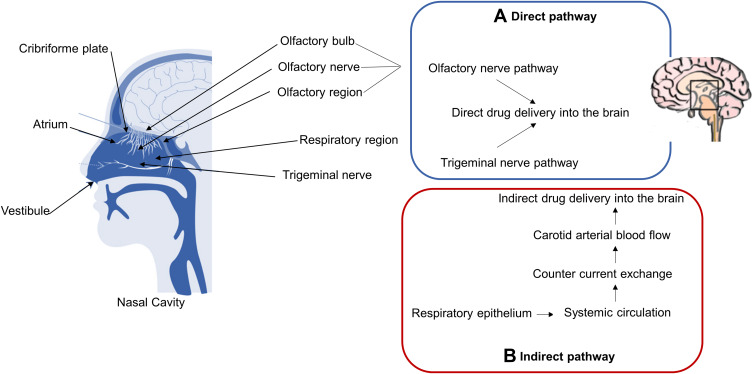
Schematic representation of nasal cavity structure and the mechanisms of drug transport through the nasal mucosa to the brain. (**A**) Direct pathway: direct drug delivery through the olfactory and trigeminal nerves. (**B**) Indirect pathway: indirect drug delivery through the countercurrent exchange mechanism in the systemic circulation.

**Notes:** Adapted from Walker KA, Ficek BN, Westbrook R. Understanding the Role of Systemic Inflammation in Alzheimer’s Disease. ACS Chem Neurosci. 2019;10(8):3340–3342.^40^ Copyright © 2019 American Chemical Society.^40^ And from Hong SS, Oh KT, Choi HG, Lim SJ. Liposomal Formulations for Nose-to-Brain Delivery: Recent Advances and Future Perspectives. Pharmaceutics. 2019;11(10):540.^114^ Creative Commons (https://creativecommons.org/licenses/by/4.0/).

The nose-to-brain delivery route is a useful and non-invasive pathway for the direct delivery of drugs into the brain.^15^ In the nasal cavity, drugs can be transported directly or indirectly to the brain, after reaching the cribriform plate (Figure 3). 76–79

#### Direct Transport

The nasal cavity and the CNS are anatomically connected by the olfactory nerve of the olfactory region and the trigeminal nerve of the respiratory region (Figure 3), which allows the direct passage of drugs to the brain.^15^,^73^,^75^ The olfactory region is considered the main region for the nose-to-brain delivery and provides a direct route that involves mechanisms of extracellular/paracellular diffusion and intracellular absorption into the olfactory neurons.^77^

##### Olfactory Nerve Pathway

After reaching the olfactory mucosa, drugs can be transported to the brain along the olfactory nerve.^74^ Drugs that interact with the ciliated olfactory receptors at the end of the olfactory neurons can be quickly transported to the CNS, passing across the cribriform plate and through the axon and the nerve bundle, reaching the olfactory bulb and the CSF.^75^ Drug transfer to the CSF and mixing with the interstitial fluid allow the drugs to be distributed into the brain.^74^,^75^,^77^ The olfactory nerve pathway is divided into intraneuronal and extraneuronal pathways, which allow the direct delivery of drugs to the brain through different transport mechanisms. In the intraneuronal pathway, the transport is along axons, while in the extraneuronal pathway, the transport occurs through perineural channels.^12^,^68^,^69^

##### Trigeminal Nerve Pathway

The trigeminal nerve connects the nasal cavity with the brain, allowing drugs to be directly delivered to the brainstem through the branches which innervate the respiratory mucosa.^14^,^74^,^79^ The drug is transported along the trigeminal nerve branches, which innervate the anterior, the dorsal part, and the lateral walls of the nasal mucosa. These branches cross the brainstem at the pons and are directed to the rest of the hindbrain and forebrain.^74^,^79^ This pathway allows for intracellular transport through the axons and extracellular transport, which includes bulk flow, diffusion through perivascular spaces, perineuronal channels, or lymphatic channels directly attached to brain tissue and CSF.^75^,^80^

#### Indirect Transport

The indirect drug transport occurs in the respiratory region and includes a countercurrent exchange of drugs in the bloodstream that may deliver high concentrations to the BBB (Figure 3). Drugs absorbed in this manner need to cross the BBB to reach the CNS, which is challenging.^74^,^77^ Although the olfactory and respiratory epithelia are rich in blood vessels, allowing the absorption of the drug into the systemic circulation,^14^,^74^,^78^ drugs may not reach the brain in therapeutic doses and are subject to elimination during their transport throughout the systemic circulation.^14^,^78^

### Nanoemulsions and Nanostructured Lipid Carriers (NLC)

The use of nanosystems, particularly lipid-based nanosystems, has been highlighted as a promising strategy to improve AD treatment.^68^,^70^ These nanosystems show advantages over conventional pharmaceutical dosage forms. For example, their lipophilic nature and small particle/droplet size facilitate drug passage through the BBB, and the encapsulation of the drug in the lipid matrix protects the formulation against enzymatic degradation, allowing active drug to reach the brain at therapeutic levels.^68^,^71^,^72^ Lipid-based nanosystems are the most suitable nanosystems for nose-to-brain delivery due to their physicochemical properties, such as particle size, hydrophobicity, and surface charge, which can be modified to enhance the drug retention time in the nasal mucosa, delay the elimination of the formulation by mucociliary clearance and, thus, enhance the amount of drug that is directly delivered into the brain.^75^,^81^

**Figure 4:**
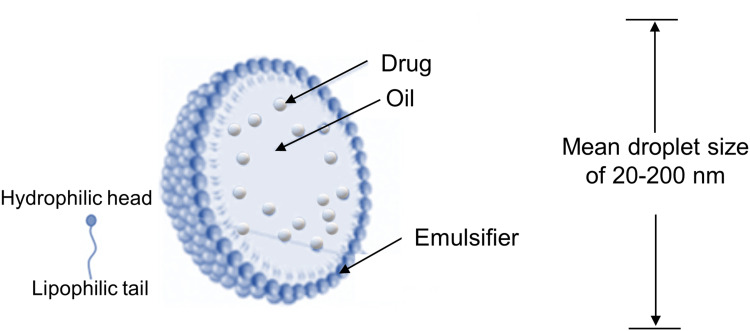
Schematic representation of the main characteristics of oil-in-water (O/W) nanoemulsions.

**Notes:** Adapted from Desfrançois C, Auzély R, Texier I. Lipid nanoparticles and their hydrogel composites for drug delivery: A review. Pharmaceuticals. 2018;11(4):118.^99^ Creative Commons (https://creativecommons.org/licenses/by/4.0/).

Nanoemulsions are nano-sized emulsions composed of two immiscible phases (water and oil) that are stabilized by one or two emulsifiers.^82–84^ There are two types of nanoemulsions, the water-in-oil (W/O) and, more commonly, oil-in-water (O/W) nanoemulsions.^85^,^86^ Typically, nanoemulsions have a mean droplet size ranging from 20 to 200 nm. Lipophilic drugs can be dissolved in the oily phase and, when the drug is released from this oily phase to the aqueous phase, nanoprecipitates can form, which have a high surface area that gives a rapid dissolution rate.^83^,^87^ Figure 4 depicts a schematic representation of the main characteristics of O/W nanoemulsions.^88^,^89^

The relevance of nanoemulsions for drug transport has increased since they have been recognized as advanced systems for targeted and controlled drug delivery.^84^,^86^ Nanoemulsions have similar advantages to other lipid-based nanosystems, such as high drug encapsulation efficiency; improved bioavailability; the possibility of sustained and targeted drug delivery; high drug absorption through several administration routes; high kinetic stability; facility for large-scale production; absence of toxicity due to the use of generally recognized as safe (GRAS) excipients.^85^,^86^

The main disadvantages of nanoemulsions are the higher amount of emulsifier required for droplets stabilization and low stability, which can be affected by pH and temperature, and during storage, due to leakage of the encapsulated drug.^86^,^90^ Besides, the low viscosity of nanoemulsion formulations makes them easy to be cleared from the nose, due to the mucociliary movement, reducing the contact time in the nasal cavity, which ultimately decreases the drug delivery into the brain.^87^,^91^ One strategy to overcome this drawback is modifying nanoemulsion with mucoadhesive polymers, which improve the formulation’s adhesion in the target site, increasing their residence time.^91^,^92^

A general review of the literature on the use of nanoemulsions for nose-to-brain delivery indicates that they have a high potential to deliver drugs directly from the nasal cavity to the brain due to their lipophilic nature, small droplet size, and high permeability through the nasal mucosa.^87^,^91^ In addition, nanoemulsions can be formulated as nanoemulgels, or in situ hydrogels to overcome the mucociliary clearance mechanism, improving residence time in the nasal cavity.^92^ Various studies have shown that nanoemulsions with small droplet size (~200 nm) and ZP close to 30 mV can deliver drugs from the nose to the brain, using dosage forms like gels and nasal sprays, which can be advantageous in the management of AD.^87^,^92^,^93^

**Figure 5:**
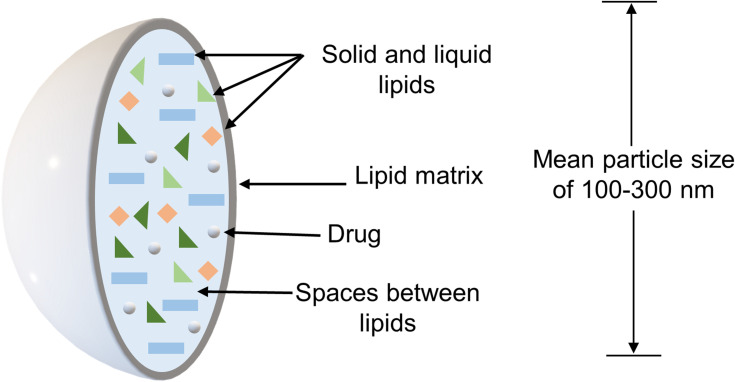
Schematic representation of the main characteristics of nanostructured lipid carriers (NLC).

Lipid nanoparticles are aqueous dispersions of solid particles and can be composed of physiological lipids and stabilized by one or two emulsifiers, which have a mean particle size that usually ranges from 100 to 300 nm, although sizes smaller than 100 nm or up to 1000 nm may also be present. There are two types of lipid nanoparticles, which are the solid lipid nanoparticles (SLN) that contain particles with a lipid matrix composed of a single solid lipid that has a highly organized inner structure, and the NLC that has a disorganized inner lipid matrix formed by a mixture of solid and liquid lipids. The disarrangement in the lipid matrix of the NLC, caused by the liquid lipid allows a higher encapsulation efficiency and low expulsion of the encapsulated drug during storage. Thus, current investigations focus on NLC.^29^,^76^,^94^ Nevertheless, some concerns related to the physical instability and safety of NLC formulations were pointed out as their main limitations. To avoid this, the stability of NLC formulations can be modified by the storage temperature and the pH.^94^,^95^ For instance, during storage, nanoparticles can aggregate, reducing the potential of NLCs as carriers for controlled drug release.^96^ Concerning safety, the determination of whether NLC can be considered as safe carriers for therapy includes the composition of the NLC formulation in terms of its biological compatibility, the effect of particle size, and surface charge.^94^,^96^ In this sense, it is essential to develop more detailed nanotoxicological studies to identify the specific elements that ensure their safety. The main characteristics of NLC are presented schematically in Figure 5.^88^,^89^

For nasal drug administration, NLC offers advantages over other nanosystems, as this nanosystem can be fabricated from biocompatible and biodegradable components, such as physiological lipids and other GRAS excipients.^78^,^95^,^97^,^98^ Moreover, NLC protects drugs against enzymatic degradation, increases residence time in the nasal cavity, and improves bioavailability. Besides, it is possible to produce NLC with the desired attributes for nose-to-brain delivery, viz., mean particle size ≤ 200 nm, PDI $\ge$ 0.3, and ZP ~30 mV.^81^,^95^,^96^,^98^ However, the low viscosity of NLC reduces the contact time of the formulation in the nasal cavity, affecting transport and drug bioavailability in the brain. To circumvent this drawback, the inclusion of mucoadhesive materials in the NLC formulations has been described as a suitable strategy.^75^,^99^ Several researchers have demonstrated the advantages of NLC for the administration of drugs used in the management of AD, which are described in section 4.5.

### In situ Hydrogels

Hydrogel formulations with suitable rheological properties are difficult to administer using a standard nasal spray device, although this can be overcome using in situ gelation.^100^ Furthermore, bioadhesive polymers can be used to increase the aptitude of formulations for nose-to-brain delivery. They can allow a more sustained release of the drug, decreasing the number of doses administered and improving the patient’s adherence to the treatment.^12^,^73^,^77^ When formulations containing mucoadhesive polymers are administered via the nasal route, the polymer chains can interact with mucin to increase the residence time of the formulation in the nasal cavity and enhance drug absorption.^12^,^77^

In situ hydrogels are formed by stimulus-sensitive polymers that, once administered to the body, undergo an in situ gelation, forming a hydrogel.^100^,^101^ Stimulus-sensitive polymers are classified according to the type of stimulus to which they respond:^102^,^103^ i) polymers that respond to biological stimuli (enzymes and biomolecules); ii) polymers that respond to chemical stimuli (pH and ionic strength); iii) polymers that respond to physical stimulus (temperature, ultrasound, light, mechanical stress).

In situ gelling hydrogels have been widely applied in the nasal administration of drugs. These formulations are administered in the form of a solution. Once in the nasal mucosa, a hydrogel is formed due to changes in the polymer’s conformation resulting from a stimulus such as pH or temperature.^68^,^100^,^101^ The use of stimulus-sensitive polymers (eg, poloxamers and poly(N-isopropyl acrylamide) in association with mucoadhesive agents, such as chitosan and hydroxypropyl methylcellulose, improves the electrostatic attractions of the formulation with the mucin present in the nasal cavity.^68^,^100^ In addition, prolonged drug delivery can be achieved if lipid-based nanosystems are included in in situ hydrogels, which provides a double protection for the drugs as they are encapsulated in the lipid matrix of the nanosystems and within the hydrogel network.^99^,^100^,^104^

### Quality by Design (QbD) Approach

In recent decades, the QbD approach has been proposed as a key element to guide manufacturers through the development of new pharmaceutical products.^18^,^105^ Accordingly, the International Council for Harmonisation (ICH) Q8 defines the QbD as a systematic approach that must be used in the development of a new pharmaceutical product based on quality risk management and focused on obtaining a final product with the desired quality target product profile (QTPP), high-quality and safety to meet customer’s needs.^18^ Thereby, the development of lipid-based nanosystems must proceed with the ICH guidelines of pharmaceutical development (Q8), quality of risk management (Q9), and pharmaceutical quality systems (Q10).^18–20^ The QbD approach determines whether a nanosystem should be designed before the manufacturing process begins employing the design of experiment (DoE). Using tools such as the Ishikawa diagram and statistical modelling, the process parameters related to the critical material attributes (CMAs) and critical process parameters (CPPs) that will ensure the critical quality attributes (CQAs) should be analyzed. Process analytical technology (PAT) is also a key parameter in the quality process, which recognizes the CQAs and CPPs that determine the final product’s quality and safety.^18^

Thus, the design of lipid-based nanosystems should consider the formulation and the device as a single entity. The CMAs related to formulations materials, such as lipid ratio and emulsifier amount should be evaluated and the CPPs associated with the production techniques should be tested. For example, different time and sonication amplitudes must be studied to obtain a final formulation with the desirable CQAs.^21^

Although there are no specific regulations for the CQAs for a nasal formulation, it is essential to consider the drug, vehicle, and the delivery device during development. Several studies have reported that a lipid-based formulation for nose-to-brain delivery should have the following CQAs: particle/droplet size lower than 200 nm; narrow PDI (between 0.2 and 0.3); zeta potential close to |30 mV|; high encapsulation efficiency (EE) ≤ (90%); controlled drug release; tonicity, viscosity, and pH adjusted to the nasal mucosa’s physiological values.^21^,^81^,^98^,^106^ Guaranteeing these CQAs, the final formulation should meet the QTPP for nasal administration and be considered safe and efficient for clinical trials, after in vitro biocompatibility studies in cell cultures, ex vivo studies in organs or tissues, and in vivo tests in animals.

### Preclinical Studies

Studies with therapeutic outcomes obtained for nanoemulsions, SLN and NLC for nose-to-brain delivery in AD treatment are summarised in Table 1.

**Table 1:** Most Relevant Therapeutic Effects of Formulations Containing Nanoemulsions and Nanostructured Lipid Carriers (NLC) for Nose-to-Brain Delivery in the Treatment of Alzheimer’s Disease (AD)

Drug-Loaded Lipid-Based Nanosystem	Excipients	Relevant Outcomes	Therapeutic Effects	Reference
Donepezil-loaded nanoemulsion	● Oil: Labrasol^®^ ● Emulsifier: Cetyl pyridinium chloride ● Co-emulsifier: Glycerol	● In vitro prolonged drug release ● Absence of cytotoxic effects	● In vivo studies revealed that nanoemulsions allowed a higher uptake of donepezil in the brain than oral and intravenous solutions of donepezil	[108]
In situ resveratrol-loaded NLC hydrogel	● Solid lipid: Cetyl palmitate ● Liquid lipid: Capmul MCM ● Emulsifier: Poloxamer 188 and Tween^®^ 80	● According to ANOVA results, the R^2^ values for particle size (0.9380), drug loading (0.8752,) and EE (0.9117) indicated that the Placket-Burman design and a two-level three-factor full factorial design were appropriated to evaluate the dependent responses ● Absence of nasal ciliotoxicity	● In vivo pharmacokinetic studies indicated higher drug distribution in the brain after intranasal administration compared to an orally administered resveratrol suspension	[68]
In situ naringenin-loaded nanoemulsion hydrogel	● Oil: Capmul MCM ● Emulsifiers: Tween^®^80 and PEG 400 (4:1) ● Polymers: Poloxame 407 and chitosan	● Ex vivo permeation studies demonstrated that drug permeated slightly more slowly from the in situ naringenin-loaded nanoemulsion hydrogel (92.72 ± 6.41% in 12h) than from the naringenin-loaded nanoemulsion (> 91.0% in 8h) ● No mortality and morphological changes in the microstructure of the brain and in the nasal mucosa	● In vivo studies showed that the in situ naringenin-loaded nanoemulsion hydrogel exhibited higher antioxidant activity and effects in the locomotion of rats compared to the respective nanoemulsion ● In situ naringenin-loaded nanoemulsion hydrogel improved the drug bioavailability in the brain after intranasal administration	[70]
Memantine-loaded nanoemulsion	● Oil: Labrasol^®^ ● Emulsifier: cetylpyridinium chloride ● Co-emulsifier: ethylene glycol and propylene glycol	● In vitro drug release of 80% in simulated nasal fluid ● Higher antioxidant potential compared to a placebo nanoemulsion cytotoxicity results showed absence of toxic effects	● In vivo pharmacokinetic studies showed higher uptake of memantine in the brain after intranasal administration compared to an aqueous and an orally and intravenously drug solution	109
Pioglitazone-loaded NLC	● Solid lipid: tripalmitin ● Liquid lipid: Capmul MCM ● Emulsifiers: Tween^®^ 80 and Pluronic^®^ F68.	● According to ANOVA results, the R^2^ values obtained for particle size (0.9807) and ZP (0.9890) showed that the Box-Behnken design was adequate to evaluate the dependent responses ● In vitro drug release from the optimized formulation exhibited Higuchi kinetic ● Higher ex vivo permeation after intranasal administration compared to a pioglitazone solution ● Absence of nasal ciliotoxicity	● In vivo biodistribution study indicated enhanced delivery of pioglitazone-loaded NLC into the brain after nasal administration	[110]
Rivastigmine hydrogen tartrate-loaded NLC	● Solid lipid: Compritol 888 ATO ● Liquid lipid: Triacetin ● Emulsifiers: Sucrose stearate and Poloxamer 188	● According to the ANOVA results, the R^2^ values for particle size (93.64%) and EE (92.47%) indicated that the three-factor and three-level Box-Behnken design was suitable to evaluate the dependent responses ● Ex vivo study showed prolonged drug release ● Aldicarb assay indicated greater drug penetration into the brain compared to a rivastigmine solution	● In vivo study indicated an improvement in memory, escape latency, and transfer latency ● Quantitative RT-PCR revealed significantly decreased acetylcholinesterase 1 and 2 expressions when compared to the same dose of a rivastigmine solution	[111]

**Abbreviations:** ANOVA, analysis of variance; EE, encapsulation efficiency; NLC, nanostructured lipid carriers; RT-PCR, reverse transcription polymerase chain reaction; ZP, zeta potential.

Fachel et al optimized a chitosan-coated rosmarinic acid-loaded nanoemulsion for nasal administration. The formulations had a mean droplet size of 225 up to 270 nm, narrow PDI, ZP greater than |20 mV|, and association efficiency close to 90%. Chitosan-coated rosmarinic acid-loaded nanoemulsion significantly reduced lipopolysaccharide-induced changes in astrocyte cell viability and decreased cell death by necrosis. The antioxidant effects were demonstrated in vitro by a decrease in ROS and nitric oxide levels and the formulation’s preventive effect in decreasing the total thiol content. It was found that chitosan-coated rosmarinic acid-loaded nanoemulsions, free rosmarinic acid, and the rosmarinic acid-loaded nanoemulsion were protective with regard to cell viability and proliferation. Besides, chitosan-coated rosmarinic acid-loaded nanoemulsions interfered with the transport of hypertrophic reactive astrocytes and regulated the astrocyte redox state. However, in vivo studies are necessary to understand the neuroprotective potential of this delivery system.^107^

Kaur et al developed nanoemulsions for the nose-to-brain delivery of donepezil, which is an AChE inhibitor used in the management of AD. Its oral administration has several limitations related to bioavailability, which can be overcome using nanoemulsions, which increase drug concentration in the brain and minimize its distribution in the periphery. The optimized formulation had a particle size of 65 nm, a PDI of 0.084, and ZP of −10.7 mV. In vitro drug release studies showed a controlled release of donepezil from the nanoemulsion. Cytotoxicity analysis indicated that the developed formulation was non-toxic. In vivo delivery studies revealed that nanoemulsions allowed greater uptake of donepezil in the brain than oral and intravenous donepezil solutions. From these findings, the authors suggested that the formulation may be an attractive approach for nose-to-brain delivery of donepezil, providing advances in the AD treatment.^108^ In another study, Kaur et al developed a nanoemulsion to improve the nose-to-brain delivery of memantine which had a particle size of ~11 nm, PDI of 0.080, and ZP of −19.6 mV. In vitro release studies demonstrated that the nanoemulsion-loaded memantine originated 80% of drug release in simulated nasal fluid, following a first-order kinetic model. Antioxidant tests indicated a higher antioxidative activity for memantine-loaded nanoemulsions than a placebo nanoemulsion. The in vivo results showed higher cell viability and fewer adverse effects with the memantine-loaded nanoemulsion than an aqueous drug solution. Biodistribution results demonstrated greater drug uptake in the brain when memantine was administered intranasally using a nanoemulsion, indicating the potential of the formulation approach to improving memantine therapeutics.^109^

Jojo et al optimized pioglitazone-loaded NLC for nose-to-brain delivery. Pioglitazone is an anti-diabetic drug with potential use in AD treatment to treat multiple targets. In preclinical models, pioglitazone significantly improved AD symptoms. However, pioglitazone’s failure in clinical trials has been associated with poor BBB penetration and peripheral adverse effects. The formulation was optimized by a Box-Behnken design, which analyzed the effects of three independent variables, viz. percentage of total emulsifier, proportion of tween^®^ 80 in the emulsifier mixture, and amount of stearyl amine on dependent responses particle size and ZP. According to the analysis of variance (ANOVA), the R^2^ values obtained for particle size (0.9807) and ZP (0.9890) showed that the design was adequate for dependent responses. The total percentage of emulsifier was inversely related to particle size and ZP, whereas the amount of stearyl amine was positively related to ZP. The predicted values of the dependent responses for an optimized formulation, particle size (208 nm) and ZP (12.5 mV), showed a close agreement with the observed values of particle size (211.4 ± 3.54 nm) and ZP (14.9 ± 1.09 mV). In vitro drug release from the optimized formulation exhibited Higuchi model kinetics. Ex vivo permeation was higher for the optimized pioglitazone-loaded NLC than a pioglitazone solution. An in vitro nasal ciliotoxicity study showed that the formulation was safe for nasal administration. In vivo biodistribution indicated enhanced delivery of pioglitazone-loaded NLC into the brain after nasal administration, demonstrating the potential of NLC as a carrier for pioglitazone in AD treatment.^110^

Anand et al developed rivastigmine hydrogen tartrate-loaded NLC for the treatment of dementia resulting from AD. Rivastigmine is an AChE inhibitor that undergoes extensive first-pass metabolism and has poor penetration of the BBB. The rivastigmine-loaded NLC was optimized using a three-factor three-level Box-Behnken design with three independent variables, sonication time, solid/lipid ratio (%), and emulsifier concentration (%) on dependent responses mean particle size and EE. ANOVA with suitable R^2^ values for particle size (93.64%) and EE (92.47%) indicated that the design was appropriated for dependent responses. The sonication time, solid/lipid ratio, and surfactant concentration had an antagonistic effect on particle size. The most significant independent variable on particle size was the sonication time (*p* value=0.001), with particle size decreasing with the increase in sonication time. Regarding EE, sonication time and emulsifier concentration showed a synergistic effect, while the solid/lipid ratio had an antagonistic effect. By using the *p*-value, the emulsifier concentration (*p*=0.004) and solid/lipid ratio (*p* value=0.002) had a more significant effect on EE. The predicted and observed values for particle size and EE were close (254 nm and 266 ± 0.94 nm, 58.95%, and 61.82 ± 2.52%, respectively). Ex vivo drug diffusion studies showed a controlled drug release from the optimized rivastigmine hydrogen tartrate-loaded NLC. An aldicarb assay demonstrated greater drug penetration into the brain compared to a rivastigmine solution. Results from quantitative real-time polymerase chain reaction (RT-PCR) revealed that rivastigmine-loaded NLC (at a dose of 400 µg) significantly decreased acetylcholinesterase 1 and 2 expressions when compared to the same dose of a rivastigmine solution. In vivo studies also indicated a significant memory improvement, escape latency, and transfer latency, suggesting that rivastigmine-loaded NLC is a promising new therapeutic approach for AD-related dementia.^111^

Cunha et al optimized two NLC formulations to direct rivastigmine from the nasal cavity to the brain. The QbD approach was used to optimize the formulations in two steps, considering the QTPP and the CQAs for intranasal administration. First, the effect of the independent variables solid/lipid and emulsifier ratio on CQAs (particle size, PDI, ZP, and EE) was analyzed through a central composite design. A second optimization was undertaken for the production method (ultrasound technique and high-pressure homogenization (HPH)), where the independent variables were revolutions per minute applied in high-speed homogenization, the amplitude of sonication, and the number of cycles used in HPH, was performed using a Box-Behnken design. According to ANOVA, the central composite design was suitable for dependent responses, since the values of R^2^ for particle size, PDI, ZP, and EE were, respectively, 0.815, 0.725, 0.932, and 0.73. The Box-Behnken design was also adequate since the R^2^ values for all dependent responses were equal to 1. The instrumental parameters that allow obtaining the best values of CQAs were selected. The most suitable rivastigmine-loaded NLC formulations prepared by ultrasound technique and HPH method had: particle size of 114.0 ± 1.9 nm and 109.0 ± 0.9 nm; PDI of 0.221 ± 0.003 and 0.196 ± 0.007; ZP of −30.6 ± 0.3 mV and −30.5 ± 0.3 mV; EE of 97.0 ± 0.5% and 97.2 ± 0.3%; pH of 6.21 ± 0.01 and 6.22 ± 0.01 and osmolarity of 279 ± 1 and 280 ± 1 mOsm/Kg. Drug release studies showed that both optimized formulations had an in vitro sustained drug release that followed a non-Fickian mechanism. Additionally, stability studies indicated that optimized rivastigmine-loaded NLC were stable after 90 days of storage. Thus, the QbD approach was used to design rivastigmine-loaded NLC with the desired QTPP for intranasal administration, which requires in vivo studies to demonstrate the preclinical efficacy and safety of these formulations.^81^

Quercetin is a flavonoid with antioxidant, anti-inflammatory, and anti-cancer activity. In AD, quercetin can reduce protein oxidation, lipid peroxidation, neuronal cell death and inhibit Aβ protein aggregation. Pinheiro et al conducted a study with quercetin encapsulated in SLN and NLC to increase quercetin’s brain bioavailability. SLN and NLC were functionalized with transferrin to promote the passage across the BBB. SLN and NLC had sizes smaller than 250 nm, ZP of ˗30 mV, and EE around 80–90%. Cytotoxicity studies performed on immortalized human cerebral microvascular endothelial cells (hCMEC/D3) confirmed an absence of SLN and NLC toxicity. The NLC promoted higher permeability through the hCMEC/D3 cells than the SLN. An in vitro model with Aβ peptide showed that quercetin-loaded NLC functionalized with transferrin decreased fibril formation and peptide aggregation when compared to a control sample. However, these findings have not yet been confirmed in vivo.^69^

Rajput et al prepared an in situ hydrogel of resveratrol-loaded NLC for intranasal administration. Resveratrol has anti-inflammatory, antioxidant, and neuroprotective effects, being useful in the prevention and treatment of AD. However, it has chemical instability and susceptibility to the first-pass metabolism. Incorporation in NLC with formulated with acyl gellan gum as an in situ gelling agent provides drug protection and mucoadhesion in the formulation of the nasal cavity. Resveratrol-loaded NLC was optimized using a Placket-Burman design that selected important independent variables related to the formulation (drug, lipid, oil, emulsifier, co-emulsifier, and solubilizer) and process parameters (probe sonication time). Afterward, a two-level three-factor full factorial design was used to study the effect of the selected independent variables viz. amount of drug, amount of emulsifier, and solubilizer on particle size, drug loading, and EE. ANOVA with R^2^ values for particle size (0.9380), drug loading (0.8752), and EE (0.9117) indicated that the design was appropriated for dependent responses. For particle size, the amount of emulsifier had the most significant effect. The drug loading increased with the increase in the amount of drug and decreased as the solubilizer concentration increases. Regarding EE, it was observed that the amount of solubilizer and drug had the most significant effects, followed by the drug and emulsifier amount. Thus, as the amount of solubilizer and drug increases, the EE decreased. The production method and materials were also optimized to obtain an in situ gel of resveratrol-loaded NLC with the desired nasal administration attribute. The final formulation had a particle size of 132 ± 12 nm, PDI of 0.165 ± 0.002, ZP of ˗23 ± 4 mV, drug loading of 10 ± 3%, and EE of 74 ± 6%. The nasal ciliotoxicity study showed that nasal tissue exposed to the in situ hydrogel of resveratrol-loaded NLC did not give rise to toxicity in the epithelial layer, basement membrane, or nuclei of glandular cells. In vivo pharmacokinetic studies in mice showed higher drug levels in the brain with the in situ hydrogel of resveratrol-loaded NLC after intranasal administration compared to an orally administered resveratrol suspension.^68^

Pires et al developed formulations of fosphenytoin, a phenytoin prodrug with neuroprotective effects, for intranasal administration. Their study aimed to obtain three different formulations: i) a nanoemulsion with faster drug release, ii) a nanoemulsion for prolonged drug release, and iii) an in situ fosphenytoin-loaded nanoemulsion hydrogel to improve the prolonged drug release and its residence time in the nasal cavity. The optimized nanoemulsions containing 90% and 60% of fosphenytoin presented a droplet size of 216.4 ± 10.5 nm and 209.2 ± 21.7 nm, a PDI of 0.305 ± 0.031 and 0.26 3 ±0.036, and a ZP of −20.8 ± 3.9 mV and −18.6 ± 0.5 mV. The in situ thermosensitive hydrogel with adequate viscosity for intranasal administration was prepared with 90% (w/w) of fosphenytoin and 17% of poloxamer 407 (%, w/v), showing a droplet size of 219.7 ± 26.8 nm, PDI of 0.237 ± 0.040, ZP of −10.7 ± 2.7 mV and osmolarity of 1375 mOsm/kg. In vitro drug release studies with the optimized formulations showed that nanoemulsions with 90% and 60% fosphenytoin had slower release than a drug solution. The in situ fosphenytoin-loaded nanoemulsion hydrogel exhibited a more prolonged drug release than the nanoemulsions. A fast drug release was achieved from nanoemulsion with 60% of fosphenytoin, which has the potential to treat acute pain episodes, while nanoemulsion with 90% fosphenytoin and in situ fosphenytoin-loaded nanoemulsion hydrogel exhibited a prolonged drug release, showing potential for the management of nasal wound healing, inflammatory reactions, and tissue remodelling.^112^ Ahmad et al developed a naringenin-loaded nanoemulsion and an in situ-based nanoemulsion hydrogel for nose-to-brain delivery to improve drug bioavailability. Naringenin is a flavonoid compound with potential for the management of AD due to its anti-inflammatory and antioxidative effects. The nanoemulsion had a droplet size of 91.39 ± 1.89 nm and a PDI of 0.372 ± 0.014. An in situ naringenin-loaded nanoemulsion hydrogel was prepared using poloxamer 407 as the gelling polymer and chitosan as the mucoadhesive agent and exhibited a droplet size of 98.31 ± 1.17 nm, a PDI of 0.386 ± 0.021, and a ZP of −19.24 mV that changed to +13.91 mV after including the nanoemulsion in the hydrogel, due to the addition of chitosan. Ex vivo permeation studies demonstrated that the drug permeated more slowly from the in situ naringenin-loaded nanoemulsion hydrogel (92.72 ± 6.41% in 12 h) than from the naringenin-loaded nanoemulsion (up to 91.0% in 8 h). In vivo biodistribution studies indicated an improvement in the bioavailability of naringenin in the brain after intranasal administration of the in situ naringenin-loaded nanoemulsion hydrogel. An in vivo evaluation of the effect of naringenin on the locomotion of rats and its antioxidant activity demonstrated better results for the in situ naringenin-loaded hydrogel when compared to the naringenin-loaded nanoemulsion. In safety studies, mortality and morphological changes in the microstructure of the brain and in the nasal mucosa of the animals were not observed. From these results, it was concluded that the naringenin-loaded nanoemulsion and its in situ-based hydrogel were safe and effective formulations to transport naringenin directly from the nasal cavity to the brain.^70^

## Conclusion and Future Prospects

The use of the nasal route is a promising alternative to parenteral and oral administration of drugs to manage AD. Nasal administration allows drugs to be transported directly from the nasal cavity to the brain, avoiding crossing the BBB. For good bioavailability of drugs in the brain, the formulations physical-chemical characteristics must be optimized for this route of transport and to avoid the physiological clearance mechanisms of the nasal cavity. Several strategies have been investigated to improve drug absorption. Lipid-based nanosystems, such as nanoemulsions and NLC, with or without in situ-forming hydrogel matrices, have been highlighted as effective approaches to achieve drug delivery from the nasal cavity to the brain. In addition, considerations for manufacturing these systems have been illustrated according to the QbD approach and the requisites for nasal administration. Recent preclinical studies have shown that nanoemulsions and NLC, and their respective in situ hydrogels, are highly promising approaches to improving the bioavailability of drugs used to treat AD in the brain using nasal administration.

However, there is a lack of uniformity in understanding the factors involving the delivery of drugs directly to the brain. Relevant factors for nasal drug delivery, including formulations’ characterization, particularly the nanosystem’s physicochemical properties (mean particle/globule size, PDI, and ZP), and accurate information about the values of osmolality, pH, and viscosity are needed. The future directions for the potential clinical use of NLC and nanoemulsions include more studies regarding their distribution, absorption, methods to upscale manufacturing process, long-term stability, more extensive in vivo studies of animal models of AD, and translation to the clinic, predicting therapeutic safety effects. Thus, these lipid-based nanosystems promise to play an essential role in the future management of AD and improve patients’ quality of life.

## Abbreviations

AD, Alzheimer’s disease; AChE, acetylcholinesterase inhibitors; NMDA, N-methyl-d-aspartate receptor antagonists; BBB, blood–brain barrier; CNS, central nervous system; NLC, nanostructured lipid carriers; EE, encapsulation efficiency; PDI, polydispersity index; ZP, zeta potential.
